# Household food insecurity is negatively associated with achievement of prenatal intentions to feed only breast milk in the first six months postpartum

**DOI:** 10.3389/fnut.2024.1287347

**Published:** 2024-01-31

**Authors:** Jane Francis, Alison Mildon, Valerie Tarasuk, Lesley Frank

**Affiliations:** ^1^Department of Sociology, Acadia University, Wolfville, NS, Canada; ^2^Department of Nutritional Sciences, University of Toronto, Toronto, ON, Canada

**Keywords:** breastfeeding, infant feeding intentions, breastfeeding intentions, food insecurity, breastfeeding disparities, pregnancy, postpartum

## Abstract

**Background:**

Household food insecurity (HFI) has been associated with suboptimal breastfeeding practices. Postpartum factors reported by caregivers include stressful life circumstances and maternal diet quality concerns. It is unknown whether prenatal breast milk feeding intentions, a well-established predictor of breastfeeding outcomes, differ by HFI status. We explored associations between HFI and prenatal intentions to feed any and only breast milk in the first 6 months postpartum, and achievement of these intentions.

**Methods:**

We utilized data from self-identified biological mothers with children 6–12 months of age who responded to a retrospective, cross-sectional online infant feeding survey conducted in Nova Scotia, Canada. HFI (yes/no) was assessed using the Household Food Security Survey Module. Prenatal intentions to feed any and only breast milk were assessed based on responses to five options for infant milk feeding plans. Achievement of intentions was assessed by breast milk and formula feeding practices in the first 6 months. Multivariable logistic regressions were conducted, adjusting for maternal socio-demographics.

**Results:**

Among 459 respondents, 28% reported HFI; 88% intended to feed any breast milk and 77% intended to feed only breast milk, with no difference by HFI status. Of those intending to feed any breast milk, 99% succeeded, precluding further analysis. Among mothers who intended to provide only breast milk, only 51% achieved their intention, with lower odds among those with HFI (aOR 0.54, 95% CI 0.29–0.98).

**Conclusion:**

HFI was not associated with intentions for feeding breast milk in the first 6 months postpartum, but mothers with HFI were less likely to achieve their intention to provide only breast milk. Further research is needed to understand the underlying reasons for this and to guide intervention designs to address HFI and help mothers reach their breastfeeding goals.

## Introduction

Household food insecurity (HFI), the insecure or inadequate access to food due to financial constraints, is a major public health concern as it represents broader material deprivation and is a strong determinant of physical and mental health for both children and adults (1–4). The most recent nationally representative Canadian data indicate that 18% of households in the ten provinces experienced food insecurity (5). This includes approximately 6.9 million individuals, including 1.8 million children under 18 years of age (5). Families with children are particularly vulnerable to food insecurity, with one in four Canadian children living in a food insecure household (5–7).

To optimize infant and maternal health and infant development, global infant feeding recommendations include exclusive breastfeeding for the first six months of life (8, 9). The most recent national Canadian data indicate that while 91% of infants initiated breastfeeding, only 35% exclusively breastfed to six months (10). As in other high-income countries, there are social disparities in breastfeeding as this behavior is negatively impacted by the social and structural determinants of health, including HFI (11–13). For instance, secondary data analysis from multiple cycles of the nationally representative, cross-sectional Canadian Community Health Survey found no difference in breastfeeding initiation based on HFI status, but respondents with HFI had lower odds of breastfeeding exclusively to four months (14). Qualitative studies reveal that mothers experiencing HFI may introduce formula or stop breastfeeding early because they are concerned about the quality and/or quantity of their breast milk due to their own inadequate dietary intake (15, 16). The perinatal period is an especially vulnerable time for some families in Canada due to increased expenses and interruptions in earnings; the national parental leave program is indexed to prior employment income up to a maximum of 55%, reducing overall household income, and low-income women may not qualify for parental leave benefits due to eligibility requirements (17, 18). The resultant stress associated with living in food insecure circumstances may negatively impact breastfeeding (16, 17, 19, 20).

Prenatal breastfeeding intentions are a strong and well-established predictor of breastfeeding outcomes (21–23). The practice of infant feeding includes mental processes and planning, but in the context of food insecurity attention has only been paid to actual postpartum feeding behaviors. To further understand the relationship between food insecurity and infant feeding and inform intervention designs, it is important to understand whether HFI status is associated with prenatal breastfeeding intentions and whether mothers experiencing HFI are able to reach their own breastfeeding goals. In this paper, we aimed to explore associations between HFI and 1) prenatal intentions to feed any breast milk in the first 6 months postpartum, 2) prenatal intentions to feed only breast milk in the first 6 months postpartum, and 3) achievement of these intentions.

## Materials and methods

### Study setting and participants

The current analysis used data from a retrospective, cross-sectional online infant feeding survey conducted within a larger multi-phased, mixed-methods study which aimed to better understand how food insecurity shapes how infants are fed. In the first phase, interviews were conducted with food insecure caregivers with children under 24 months to investigate how they navigate feeding their infants on a daily basis. Second, based on the interview findings and prior qualitative research, infant food insecurity indicators were created. A larger online survey was created including these infant food insecurity indicators alongside a variety of questions related to infant feeding practices, household food insecurity, and socio-demographic characteristics. Before the survey was launched in the third phase of research, it was pre-tested among 20 caregivers with a child under 24 months of age to ensure the questions were understandable and acceptable.

Any primary caregiver with at least one child between 0–24 months of age who was living in Nova Scotia, Canada was eligible to complete the survey. Primary caregivers were defined as those who were primarily responsible for caring, raising, and feeding the child, and participants selected their caregiving role (e.g., biological mother or father, adoptive mother or father, foster mother or father, grandparent, etc.). To ensure representation of food insecure families in the survey, targeted recruitment was conducted whereby postcards with the survey information were distributed to caregivers through the 25 Family Resource Centers across the province. Family Resource Centers are non-profit, community-based organizations that provide programming and services to families with minimal resources and who are negatively impacted by the social determinants of health. An electronic version of the study postcard was posted in Family Resource Center Facebook groups, when available. For general recruitment, electronic postcards were also posted online through paid Facebook advertisements and in approximately 10 relevant Facebook groups for families of young children in Nova Scotia.

The sample for the current analysis was drawn from respondents who identified their caregiving role as the biological mother. The Household Food Security Survey Module, used to assess HFI status and described in more detail below, has a 12-month recall period; therefore, only survey respondents with infants 6–12 months of age were included in the analytic sample to ensure HFI status reflected the infant feeding period of interest (e.g., the first six months postpartum). Data were excluded from participants with no HFI data, multiple births, or preterm birth.

All surveys were completed between January and April 2022. Participants provided consent online prior to starting the survey.

### Data collection and measures

The survey was conducted using the Acadia University Survey System platform and was self-administered; therefore, all data were self-reported by participants.

Participants completed the validated 18-item Household Food Security Survey Module, which assesses the presence or absence of household food insecurity as well as the severity (none [secure], marginal, moderate, or severe) based on the number of affirmative responses (24). All questions within the Household Food Security Survey Module have a 12-month recall period.

Participants were asked about their prenatal plan for feeding their child in the first six months postpartum (breast milk only, fed directly at the breast; breast milk only, some amount of pumping; mixed feeding of breast milk and formula; formula only; or no infant feeding intention). Participants reporting any of the first three of these choices were classified as intending to feed any breast milk. Participants reporting either of the first two choices were classified as intending to feed only breast milk.

Among participants who intended to feed any amount of breast milk, achievement of this intention was determined by an affirmative response to the question assessing breastfeeding initiation, “Was the child ever breastfed or given breast milk?.” Among those who intended to feed only breast milk, achievement of this intention was determined by the participant reporting initiating breastfeeding and not introducing formula before their infant was six months of age.

Among participants classified as intending to feed only breast milk, prenatal intended mode of breast milk delivery (e.g., only at the breast or some amount of pumping) was also assessed since pumping early in the postpartum period has been associated with early cessation of any and exclusive breast milk feeding (25–27). The survey did not collect data on actual mode of breast milk delivery postpartum.

The assessment of feeding intentions and their achievement was only related to milk feeds (e.g., breast milk and formula), consistent with other literature on breast milk feeding intentions (28, 29). Therefore, data on feeding only breast milk cannot be interpreted as following the World Health Organization definition of exclusive breastfeeding, which requires consideration of all types of feeds (9).

Socio-demographic characteristics included as confounders based on availability from the survey and considered to be associated with both HFI and breastfeeding intentions/practices included single parenting (yes, no), highest level of completed education (high school or less, postsecondary), geographic location (urban, rural), parity (primiparous, multiparous), annual household income before tax (low [<$10,000–$39,999], medium [$40,000–$79,999], high [$80,000- ≥$150,000]), and age (19–27, 28–36, 37–43 years) (5, 10, 11, 30–32).

### Statistical analysis

Frequencies and percentages were calculated for socio-demographic characteristics, breast milk feeding intentions, and achievement of intentions. Chi-square tests were conducted to assess sociodemographic differences by HFI status.

Logistic regression analyses were conducted to investigate the associations between HFI status as the independent variable and three separate outcome variables: (1) intention to feed any breast milk; (2) intention to feed only breast milk; and (3) achievement of intention to feed only breast milk. Almost every participant (99%) with prenatal intention to feed any breastmilk achieved this, so no further analysis was conducted for this outcome. Two regression models were run for each of the three outcome variables: an unadjusted model and a model adjusted for the abovementioned socio-demographic characteristics. Results are presented as odds ratios (OR), 95% confidence intervals (CI), and *p* values. A p value <0.05 was considered statistically significant.

Small sample sizes within each HFI category (marginal, moderate, or severe) precluded analysis of socio-demographic characteristics, breast milk feeding intentions, and achievement of intentions by severity of HFI.

Frequencies and percentages were calculated, and a chi square test was performed, to assess achievement of intention to feed only breast milk by prenatal intended mode of breast milk delivery (e.g., only at the breast or some amount of pumping). For the outcome variable ‘achievement of intention to feed only breast milk’, an additional logistic regression model was conducted including prenatal intended mode of breast milk delivery as well as all socio-demographic characteristics.

Statistical multicollinearity among all independent variables was assessed prior to modeling using variance inflation factor (>2.5) and none was identified. Goodness of fit for each multivariable model was assessed using the Hosmer Lemeshow test (*p* > 0.05). IBM SPSS Statistics for Windows, Version 29 (IBM Corp, Armonk, New York, USA) was used to perform all analyses.

## Results

### Study participants

**Figure 1:**
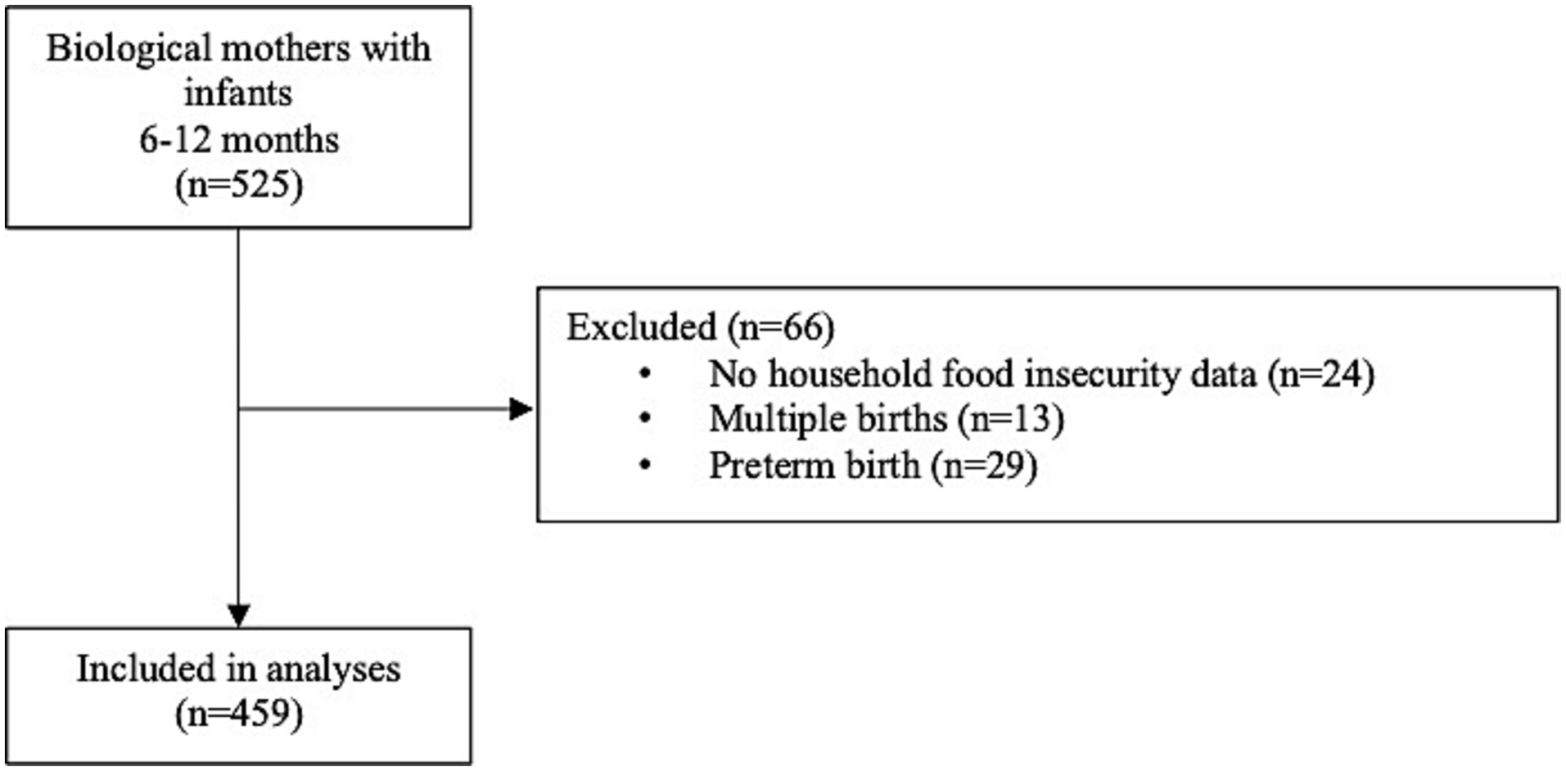
Participant flow diagram.

Overall, 525 survey respondents identified as biological mothers and had infants 6–12 months of age (Figure 1). Of these, 66 were excluded due to missing HFI data (n = 24), multiple births (n = 13), and preterm birth (n = 29). For excluded participants, there was no difference based on HFI status for multiple births, but respondents with HFI were more likely to have preterm births (Supplementary Table S1). In total, 459 participants were included in the study.

The prevalence of HFI was 28% (Table 1). Few participants were single parenting (7%), half were primiparous (51%), and almost half had annual household incomes less than $80,000 (44%). All socio-demographic characteristics differed by HFI status (*p* < 0.05), except for geographic location. For example, a higher proportion of HFI respondents were single parents, had high school education or less, were multiparous, had lower household incomes, and were younger in age.

**Table 1:** Maternal characteristics of the study sample.

Characteristic	Total sample *n* (%)	Food secure *n* (%)	Food insecure *n* (%)	*p* value^a^
**Household food insecurity status (n = 459)**				
Food insecure	127 (27.7)	N/A		
Marginal	45 (9.8)			
Moderate	55 (12.0)			
Severe	27 (5.9)			
Food secure	332 (72.3)			
**Education (n = 430)**				
High school education or less	63 (14.7)	35 (11.1)	28 (24.1)	**<0.001**
Postsecondary education	367 (85.3)	279 (88.9)	88 (75.9)	
**Parity (n = 430)**				
Primiparity	218 (50.7)	173 (55.1)	45 (38.8)	**0.003**
Multiparity	212 (49.3)	141 (44.9)	71 (61.2)	
**Single parent (n = 428)**				
Yes	29 (6.8)	10 (3.2)	19 (16.7)	**<0.001**
No	399 (93.2)	304 (96.8)	95 (83.3)	
**Geographic location (n = 412)**				
Rural	131 (31.8)	94 (30.6)	37 (35.2)	0.380
Urban	281 (68.2)	213 (69.4)	68 (64.8)	
**Household income (n = 425)**				
Low (<$10,000 to $39,999)	69 (16.2)	25 (8.0)	44 (38.6)	**<0.001**
Medium ($40,000 to $79,999)	119 (28.0)	75 (24.1)	44 (38.6)	
High ($80,000 to ≥$150,000)	237 (55.8)	211 (67.8)	26 (22.8)	
**Age in years (n = 421)**				
19–27	84 (20.0)	52 (16.8)	32 (28.6)	**0.026**
28–36	288 (68.4)	221 (71.5)	67 (59.8)	
37–43	49 (11.6)	36 (11.7)	13 (11.6)	

a Pearson chi square test.

Denominators differ for each sociodemographic characteristic due to missing data.

### Prenatal breast milk feeding intentions

Overall, 88% of participants intended to feed any breast milk (Table 2). Among food secure participants, 91% intended to feed any breast milk compared to 80% of those who were food insecure. Seventy-seven percent of all participants intended to feed only breast milk, with 80% of food secure participants intending to do so versus 69% of food insecure participants.

**Table 2:** Breast milk feeding intentions in the first six months postpartum by household food insecurity status.

	Total (*N* = 459) *n* (%)	Food secure (*N* = 332)n (%)	Food insecure (*N* = 127) n (%)	*p* value^a^
**Intention to feed any breast milk**				
Yes	403 (87.8)	301 (90.7)	102 (80.3)	**0.002**
No	56 (12.2)	31 (9.3)	25 (19.7)	
**Intention to feed only breast milk**				
Yes	352 (76.7)	265 (79.8)	87 (68.5)	**0.010**
No	107 (23.3)	67 (20.2)	40 (31.5)	

a Pearson chi square test.

In unadjusted analyses (Table 3), food insecure participants had lower odds of intending to feed any breast milk (OR 0.42, 95% CI 0.24–0.75) and only breast milk (OR 0.55, 95% CI 0.35–0.87) compared to food secure participants. However, HFI status was no longer statistically significant in the adjusted models, in which household income was the only statistically significant predictor of intention to feed any and only breast milk. Compared to participants with medium household incomes, those with low incomes had 54% lower odds of intending to feed only breast milk (95% CI 0.22–0.97). Participants with high incomes had 3 times higher odds of intending to feed any breast milk (95% CI 1.43–7.48) and almost 2 times higher odds of intending to feed only breast milk (95% CI 1.10–3.53) than those with medium incomes.

**Table 3:** Logistic regression results: associations between household food insecurity status and breast milk feeding intentions in the first six months postpartum.

	Intention to feed any breastmilk				Intention to feed only breastmilk			
	Unadjusted OR (95% CI)^a^	*p* value	Model 1 OR (95% CI)^b^^,^^c^^,^^d^	*p* value	Unadjusted OR (95% CI)^a^	*p* value	Model 1 OR (95% CI)^b^^,^^c^^,^^e^	*p* value
**Household food insecurity status**								
Food secure	1.00 (ref)	**0.003**	1.00 (ref)	0.492	1.00 (ref)	**0.011**	1.00 (ref)	0.512
Food insecure	0.42 (0.24–0.75)		0.77 (0.37–1.62)		0.55 (0.35–0.87)		0.82 (0.45–1.48)	
**Education**								
Postsecondary graduation	N/A	N/A	1.00 (ref)	0.537	N/A	N/A	1.00 (ref)	0.165
High school graduation or less	N/A		1.34 (0.53–3.37)		N/A		1.75 (0.80–3.85)	
**Parity**								
Primiparity	N/A	N/A	1.00 (ref)	0.273	N/A	N/A	1.00 (ref)	0.271
Multiparity	N/A		0.67 (0.33–1.37)		N/A		0.75 (0.45–1.25)	
**Single parent**								
No	N/A	N/A	1.00 (ref)	0.176	N/A	N/A	1.00 (ref)	0.254
Yes	N/A		2.57 (0.66–10.14)		N/A		1.87 (0.64–5.46)	
**Geographic location**								
Urban	N/A	N/A	1.00 (ref)	0.600	N/A	N/A	1.00 (ref)	0.782
Rural	N/A		0.83 (0.41–1.67)		N/A		1.08 (0.63–1.85)	
**Household income**								
Low (<$10,000 to $39,999) vs. Medium ($40,000 to $79,999) [ref]	N/A	N/A	0.46 (0.20–1.13)	**<0.001**	N/A	N/A	0.46 (0.22–0.97)	**0.001**
High ($80,000 to ≥$150,000) vs. Medium ($40,000 to $79,999) [ref]	N/A		3.27 (1.43–7.48)		N/A		1.97 (1.10–3.53)	
Low (<$10,000 to $39,999) vs. High ($80,000 to ≥$150,000)	N/A		0.15 (0.05–0.40)		N/A		0.23 (0.11–0.51)	
**Age in years**								
28–36	N/A	N/A	1.00 (ref)	0.192	N/A	N/A	1.00 (ref)	0.263
19–27	N/A		0.48 (0.22–1.06)		N/A		0.70 (0.36–1.34)	
37–43	N/A		0.89 (0.30–2.63)		N/A		0.60 (0.29–1.23)	

a Sample size for unadjusted models *n* = 459.

b Sample size for adjusted models *n* = 399.

c Models adjusted for maternal education, parity, single parenting, geographic location, household income, and age.

d Hosmer and Lemeshow *p* = 0.030.

e Hosmer and Lemeshow *p* = 0.659.

The proportion of participants in each of the five prenatal infant feeding intention categories and differences by HFI status are reported in Supplementary Table S2. Among all participants, a higher proportion of food insecure compared to food secure participants had no prenatal infant feeding plan (*p* = 0.013), while a higher proportion of food secure compared to food insecure participants intended to provide breast milk only with some amount of pumped milk (*p* = 0.043). Among the subset of participants who intended to feed only breast milk, there was no difference in the intended mode of breast milk delivery (e.g., at the breast vs. some amount of pumping) by HFI status (Supplementary Table S3).

### Achievement of breast milk feeding intentions

Among those who intended to feed any breast milk, 99% achieved their intention by initiating breast milk feeding (Table 4). Among participants who intended to feed only breast milk, 51% achieved this intention in the first six months postpartum; 49% of participants provided both breast milk and formula in the first six months postpartum, and 1% provided only formula (Table 4). Among food secure participants, 52% achieved their intention to feed only breast milk versus 48% of food insecure participants.

**Table 4:** Achievement of intention to feed any and only breast milk in the first six months postpartum.

	Intended to feed any breast milk		Intended to feed only breast milk			
	Achieved intention	Did not achieve intention		Achieved intention	Did not achieve intention	
	Initiated breast milk feeding *n* (%)	Did not initiate breast milk feeding *n* (%)		Only fed breast milk *n* (%)	Breast milk and formula *n* (%)	Only formula *n* (%)
Total (N = 403)	398 (98.8)	5 (1.2)	Total (N = 352)	178 (50.6)	171 (48.6)	3 (0.8)
Food Secure (N = 301)	297 (98.7)	4 (1.3)	Food Secure (N = 265)	137 (51.7)	126 (47.5)	2 (0.8)
Food Insecure (N = 102)	101 (99.0)	1 (1.0)	Food Insecure (N = 87)	41 (47.1)	45 (51.7)	1 (1.1)

There was no association between HFI status and achievement of intention to feed only breast milk in the unadjusted logistic regression model (Table 5). However, in the model adjusted for socio-demographic characteristics, food insecure participants had lower odds of achieving this intention (OR 0.54, 95% CI 0.29–0.98) compared to food secure participants. Parity was also statistically significant; compared to primiparous participants, those who were multiparous had higher odds of achieving their intention to provide only breast milk (OR 2.13, 95% CI 1.31–3.44).

**Table 5:** Logistic regression results: association between household food insecurity status and achievement of intention to feed only breastmilk in the first six months postpartum.

	Achievement of intention to feed only breastmilk for first 6 months postpartum			
	Unadjusted OR (95% CI)^a^	*p* value	Model 1^b^^,^^c^^,^^d^	*p* value
**Household food insecurity status**				
Food secure	1.00 (ref)	0.460	1.00 (ref)	**0.043**
Food insecure	0.83 (0.51–1.35)		0.54 (0.29–0.98)	
**Education**				
Postsecondary graduation	N/A	N/A	1.00 (ref)	0.811
High school graduation or less	N/A		0.91 (0.43–1.93)	
**Parity**				
Primiparity	N/A	N/A	1.00 (ref)	**0.002**
Multiparity	N/A		2.13 (1.31–3.44)	
**Single parenting**				
No	N/A	N/A	1.00 (ref)	0.431
Yes	N/A		0.62 (0.18–2.06)	
**Geographic location**				
Urban	N/A	N/A	1.00 (ref)	0.259
Rural	N/A		0.75 (0.45–1.24)	
**Household income**				
Low (<$10,000 to $39,999) vs. Medium ($40,000 to $79,999) [ref]	N/A	N/A	0.65 (0.27–1.60)	0.382
High ($80,000 to ≥$150,000) vs. Medium ($40,000 to $79,999) [ref]	N/A		0.70 (0.40–1.22)	
Low (<$10,000 to $39,999) vs. High ($80,000 to ≥$150,000)	N/A		0.94 (0.38–2.31)	
**Age in years**				
28–36	N/A	N/A	1.00 (ref)	0.509
19–27	N/A		1.38 (0.72–2.66)	
37–43	N/A		0.81 (0.38–1.74)	

a Sample size for unadjusted model *n* = 352.

b Sample size for adjusted models *n* = 311.

c Model adjusted for maternal education, parity, geographic location, household income, single parenting, and age.

d Hosmer Lemeshow test *p* = 0.478.

When considering intended mode of breast milk delivery, a higher proportion of participants who achieved their intention to feed only breast milk intended to feed only at the breast versus providing some amount of pumped milk (*p* < 0.001; Supplementary Table S4). When investigating the association between HFI status and achievement of intention to feed only breast milk while adjusting for prenatal intended mode of breast milk delivery as well as all socio-demographic characteristics, the relationship was strengthened (OR 0.46, 95% CI 0.24–0.87; Supplementary Table S5). In addition to parity remaining statistically significant, participants who planned to provide some amount of pumped milk had lower odds of achieving their intention to provide only breast milk (OR 0.25, 95% CI 0.15–0.41) compared to those who planned to only feed at the breast.

## Discussion

In this study, we investigated associations between HFI and prenatal intentions to feed any and only breast milk in the first six months postpartum, and achievement of these intentions. We found no difference in breast milk feeding intentions by HFI status. Achievement of intentions was very high for providing any breast milk, but only 51% for providing only breast milk, with lower odds among participants with HFI.

To our knowledge, this is the first study to investigate associations between HFI, prenatal breastfeeding intentions, and achievement of intentions. Breast milk feeding intentions among the group of mothers in this study are consistent with the breastfeeding initiation rate for the Atlantic region of Canada (81%), of which Nova Scotia is a part (10). No national data are collected on breastfeeding intentions; therefore, it is unknown how intentions from this cohort compare to the Atlantic region or to Canada as a whole. Since breastfeeding intentions are modifiable and are associated with later breastfeeding practices, it would be beneficial for national and cohort studies in Canada to collect intentions as part of their infant feeding data (33, 34). This would allow for the investigation of associations between relevant social determinants of health and breast milk feeding intentions to further understand the relationship between prenatal intentions and the social disparities in Canadian breastfeeding practices (10, 11).

In the current study, there was no difference in prenatal intentions based on HFI status. This suggests that postpartum experiences (which include the interplay of individual, interpersonal, community, and societal factors) may contribute to our finding of lower odds of achieving intentions to feed only breast milk, and the lower breastfeeding rates that several others have found among those who are food insecure (12, 14, 19, 35, 36). This merits exploration in future studies, alongside the role that household income may have on prenatal breastfeeding intentions, regardless of HFI status, since income was found to be the only predictor of intentions in adjusted analyses.

It is encouraging that almost every participant in this study who intended to feed any breast milk achieved this by initiating breast milk feeding. It is concerning that overall there was a lack of attainment of intention to feed only breast milk, as only half of the participants who wanted to provide only breast milk for the first six months postpartum were able to do so. These findings align with previous evidence that a high proportion of women across the socio-economic spectrum do not meet their breastfeeding goals and introduce other foods or stop breastfeeding earlier than they want to (28, 32, 37, 38). This underscores the need to strengthen support for all women so that they can achieve their breastfeeding goals, including those who are food insecure (39).

Among mothers with HFI in our study, there was an increased likelihood of not meeting intentions to feed only breast milk. We did not assess reasons for this, but it would be important for future studies to investigate whether the challenges among food insecure women align with those reported in the general population. Although information on breastfeeding intentions or meeting intentions is not available in the Canadian Community Health Survey, our results are consistent with analyses from this nationally-representative dataset which indicate that mothers with HFI were no less likely to start breastfeeding than those without HFI, but they were more likely to stop exclusively breastfeeding earlier (14). Together, these findings reinforce the breastfeeding paradox, that families who can least afford infant formula are the most likely to use it, and highlight that HFI may be an important and underrecognized social determinant of infant feeding (40).

Our finding that HFI was associated with lower odds of achieving intentions to feed only breast milk has potential implications for food insecure families, and for infant food insecurity specifically. Infant food insecurity refers to infant vulnerability with respect to food access, sub-optimal quality, and inadequate quantity due to household financial constraints (16). Non-exclusive breast milk feeding requires infants to receive infant formula, which is an insecure food system for low-income families due to its high cost (15, 16, 20). Qualitative research has found that breastfeeding can be a food security measure for low-income families so that they do not have to worry about purchasing infant formula, but breastfeeding can also be an insecure food system since mothers themselves perceive that the amount and quality of their breast milk is inadequate due to their own poor dietary intake (15, 16, 19, 41–43). To our knowledge, the relationship between maternal or household food insecurity and breast milk volume or composition has not been studied in high-income countries. Research in Nova Scotia found that income assistance and maternity benefits based on minimum wage earnings would not allow families to purchase a basic nutritious diet regardless if their infant was exclusively formula-fed or breastfed, compromising the nutrition of the entire household (17). In the Canadian province of Manitoba, a modest unconditional prenatal income supplement for low-income women was associated with improved breastfeeding initiation, but longer-term breastfeeding outcomes were not assessed and the income supplement did not continue in the postpartum period (44, 45). Thus, there are potential opportunities to reduce breastfeeding disparities and support the health and nutrition of families with young children through improved income supports.

As part of our exploratory analyses, we found that prenatal intended mode of breast milk delivery was an independent predictor of meeting intentions to feed only breast milk. Specifically, intentions to feed some amount of pumped milk compared with feeding only at the breast were associated with lower odds of achieving intentions to provide only breast milk. There is limited literature on pumping intentions, but studies suggest that anticipation of breastfeeding difficulties and concerns about breast milk supply are two main concerns associated with plans to pump and early pump use, which aligns with evidence that pumped/expressed breast milk feeding practices are negatively associated with longer-term breastfeeding outcomes (26, 27, 46–48). As the use of expressed breast milk may reflect lactation difficulties and reduced breastfeeding self-efficacy, this reinforces global guidance that skilled lactation support be available and accessible to all as a standard of care in the early postpartum period (26, 46, 49). We did not collect data on breastfeeding self-efficacy (50) or actual mode of breast milk delivery postpartum, therefore further research is needed to understand associations between pumping intentions, practices, and achievement of breastfeeding goals, and the role of breastfeeding self-efficacy.

Strengths of this study include successful targeted recruitment to ensure representation of food insecure caregivers in the study. The prevalence of HFI in this study (28%) was slightly higher than the national rate for households with children (20%) (5). This enabled investigation of differences in breast milk feeding intentions and achievement of intentions by HFI status. However, our sample was insufficient to support an analysis of breast milk feeding intentions and achievements by severity of HFI. Other strengths include the use of the validated 18-item Household Food Security Survey Model and detailed data collection on the provision of breast milk and formula. The retrospective nature of the survey may have introduced recall bias regarding timing of the introduction of formula, but we used all available data on formula use and the median infant age at survey completions was 9 months. There are additional limitations of this work to consider. First, we did not ask participants at what point in their pregnancy they developed their infant feeding intentions, therefore, HFI status and prenatal intentions may not be concurrent. However, all participants in the analytic sample had infants 6–12 months of age (the median infant age at survey completion was 9 months, as mentioned above) and the Household Food Security Module had a 12-month recall period. As such, a portion of the prenatal period would be captured in most participants’ food insecurity responses. Additionally, a recent Canadian study that collected HFI in the pre and postnatal period found that the HFI status of 80% of participants remained the same over both time periods (51). Second, although the survey was pre-tested to ensure questions were understandable and acceptable, the survey was self-administered, therefore, participants could not ask for clarity or more detail on survey questions. Third, as data were self-reported, there is the risk of social desirability bias. Fourth, the survey was only available online and in English, which could have precluded participation for some caregivers. Fifth, the survey was part of exploratory research to better understand how food insecurity shapes how infants are fed. As such, there was a relatively small sample size for multivariable analyses. In addition, the results are based on a convenience sample and are not population-based and not generalizable beyond groups similar to the study sample.

## Conclusion

In summary, HFI was not associated with intentions for feeding breast milk in the first 6 months postpartum, but mothers with HFI were less likely to achieve their intention to provide only breast milk. This suggests that mothers experiencing HFI encounter additional challenges that impede exclusive breastfeeding and that differences in achievement of breast milk feeding intentions between food insecure and food secure households may contribute to breastfeeding disparities. Further research is needed to understand the underlying reasons for these differences and to guide intervention designs to address HFI and help mothers reach their breastfeeding goals.

## Data availability statement

The datasets presented in this article are not readily available because of participant confidentiality and privacy. Requests to access the datasets should be directed to LF, lesley.frank@acadiau.ca.

## Ethics statement

The studies involving humans were approved by Acadia University Research Ethics Board (19-35) and the University Research Ethics Board at Mount Saint Vincent University (2019-067). The studies were conducted in accordance with the local legislation and institutional requirements. The participants provided their written informed consent to participate in this study.

## Author contributions

JF: Conceptualization, Data curation, Formal analysis, Investigation, Methodology, Project administration, Writing – original draft. AM: Formal analysis, Writing – review & editing, Methodology. VT: Methodology, Writing – review & editing, Formal analysis. LF: Conceptualization, Data curation, Funding acquisition, Investigation, Methodology, Resources, Supervision, Writing – review & editing, Formal analysis.
