# Supplementary material for: Household food insecurity is negatively associated with achievement of prenatal intentions to feed only breast milk in the first six months postpartum
Source: Front Nutr. 2024 Jan 31;11:1287347. doi: 10.3389/fnut.2024.1287347 (PMC10865492; doi:10.3389/fnut.2024.1287347)
Supplement: Supplementary file 1 [file Table_1.DOCX]

**Supplementary Table 1.** Comparison of exclusion criteria characteristics and household food insecurity status

|  | **Food Secure, n (%)** | **Food Insecure, n (%)** | **P value^a^** |
| --- | --- | --- | --- |
| **Multiple birth (n=501)** | | | |
| Yes | 8 (2.2) | 5 (3.4) | 0.536 |
| No | 348 (97.8) | 140 (96.6) |  |
| **Preterm birth (n=495)** | | | |
| Yes | 15 (4.3) | 13 (9.0) | **0.038** |
| No | 336 (95.7) | 131 (91.0) |  |

^a^ Pearson chi square test or Fisher’s exact test
