# Supplementary material for: Household food insecurity is negatively associated with achievement of prenatal intentions to feed only breast milk in the first six months postpartum
Source: Front Nutr. 2024 Jan 31;11:1287347. doi: 10.3389/fnut.2024.1287347 (PMC10865492; doi:10.3389/fnut.2024.1287347)
Supplement: Supplementary file 2 [file Table_2.DOCX]

**Supplementary Table 2.** Prenatal infant feeding intentions among all participants (n=459)

|  | **Total sample**  **n (%)** | **Food Secure**  **n (%)** | **Food Insecure**  **n (%)** | **P value^a^** |
| --- | --- | --- | --- | --- |
| Breast milk only, fed directly at the breast | 162 (35.3) | 118 (35.5) | 44 (34.6) | 0.857 |
| Breast milk only, some amount of pumping | 190 (41.4) | 147 (44.3) | 43 (33.9) | **0.043** |
| Mixed feeding of breast milk and formula | 51 (11.1) | 36 (10.8) | 15 (11.8) | 0.768 |
| Formula only | 37 (8.1) | 22 (6.6) | 15 (11.8) | 0.068 |
| No prenatal infant feeding plan | 19 (4.1) | 9 (2.7) | 10 (7.9) | **0.013** |

^a^ Pearson chi square test or Fisher’s exact test
