# Supplementary material for: Household food insecurity is negatively associated with achievement of prenatal intentions to feed only breast milk in the first six months postpartum
Source: Front Nutr. 2024 Jan 31;11:1287347. doi: 10.3389/fnut.2024.1287347 (PMC10865492; doi:10.3389/fnut.2024.1287347)
Supplement: Supplementary file 3 [file Table_3.DOCX]

**Supplementary Table 3.** Intended mode of breast milk delivery among subset of participants who intended to feed only breast milk for first six months (n=352)

|  | **Total sample**  **n (%)** | **Food Secure**  **n (%)** | **Food Insecure**  **n (%)** | **P value^a^** |
| --- | --- | --- | --- | --- |
| Intended mode of breast milk delivery | | | | |
| Breast milk only, fed directly at the breast | 162 (46.0) | 118 (44.5) | 44 (50.6) | 0.326 |
| Breast milk only, some amount of pumping | 190 (54.0) | 147 (55.5) | 43 (49.4) |  |

^a^ Pearson chi square test
