# Supplementary material for: Household food insecurity is negatively associated with achievement of prenatal intentions to feed only breast milk in the first six months postpartum
Source: Front Nutr. 2024 Jan 31;11:1287347. doi: 10.3389/fnut.2024.1287347 (PMC10865492; doi:10.3389/fnut.2024.1287347)
Supplement: Supplementary file 4 [file Table_4.DOCX]

**Supplementary Table 4.** Achievement of intention to feed only breast milk by prenatal intended mode of breast milk delivery

|  | **Intended to feed only breast milk** | | |
| --- | --- | --- | --- |
|  | Intended to feed only at the breast  n/N (%) | Intended to do some pumping  n/N (%) | P value^a^ |
| Total | 162/352 (46.0) | 190/352 (54.0) |  |
| Achieved intention to feed only breast milk | 106/178 (59.6) | 72/178 (40.4) | **<0.001** |
| Did not achieve intention to feed only breast milk | 56/174 (32.2) | 118/174 (67.8) |  |

^a^ Pearson chi square test
