# Supplementary material for: Household food insecurity is negatively associated with achievement of prenatal intentions to feed only breast milk in the first six months postpartum
Source: Front Nutr. 2024 Jan 31;11:1287347. doi: 10.3389/fnut.2024.1287347 (PMC10865492; doi:10.3389/fnut.2024.1287347)
Supplement: Supplementary file 5 [file Table_5.DOCX]

**Supplementary Table 5.** Logistic regression results: association between household food insecurity status and achievement of intention to feed only breast milk in the first six months postpartum

|  | **Achievement of intention to feed only breastmilk for first 6 months postpartum** | | | | | |
| --- | --- | --- | --- | --- | --- | --- |
|  | Unadjusted OR (95% CI)^a^ | P value | Model 1^b,c,d^ | P value | Model 2 ^b,e,f^ | P value |
| **Household food insecurity status** | | | | | | |
| Food secure | 1.00 (ref) | 0.460 | 1.00 (ref) | **0.043** | 1.00 (ref) | **0.017** |
| Food insecure | 0.83 (0.51-1.35) |  | 0.54 (0.29-0.98) |  | 0.46 (0.24-0.87) |  |
| **Education** | | | | | | |
| Postsecondary graduation | N/A | N/A | 1.00 (ref) | 0.811 | 1.00 (ref) | 0.946 |
| High school graduation or less | N/A |  | 0.91 (0.43-1.93) |  | 0.97 (0.44-2.14) |  |
| **Parity** | | | | | | |
| Primiparity | N/A | N/A | 1.00 (ref) | **0.002** | 1.00 (ref) | **0.012** |
| Multiparity | N/A |  | 2.13 (1.31-3.44) |  | 1.91 (1.15-3.17) |  |
| **Single parenting** | | | | | | |
| No | N/A | N/A | 1.00 (ref) | 0.431 | 1.00 (ref) | 0.752 |
| Yes | N/A |  | 0.62 (0.18-2.06) |  | 0.81 (0.23-2.93) |  |
| **Geographic location** | | | | | | |
| Urban | N/A | N/A | 1.00 (ref) | 0.259 | 1.00 (ref) | 0.196 |
| Rural | N/A |  | 0.75 (0.45-1.24) |  | 0.70 (0.41-1.20) |  |
| **Household income** | | | | | | |
| Low (<$10,000 to $39,999) vs Medium ($40,000 to $79,999) [ref] | N/A | N/A | 0.65 (0.27-1.60) | 0.382 | 0.61 (0.23-1.57) | 0.416 |
| High ($80,000 to ≥$150,000) vs Medium ($40,000 to $79,999) [ref] | N/A |  | 0.70 (0.40-1.22) |  | 0.71 (0.39-1.29) |  |
| Low (<$10,000 to $39,999) vs High ($80,000 to ≥$150,000) [ref[ | N/A |  | 0.94 (0.38-2.31) |  | 0.85 (0.33-2.2) |  |
| **Age in years** | | | | | | |
| 28-36 | N/A | N/A | 1.00 (ref) | 0.509 | 1.00 (ref) | 0.514 |
| 19-27 | N/A |  | 1.38 (0.72-2.66) |  | 1.34 (0.67-2.66) |  |
| 37-43 | N/A |  | 0.82 (0.38-1.76) |  | 0.74 (0.33-1.70) |  |
| **Intended mode of breast milk delivery** | | | | | | |
| Only at the breast | N/A | N/A | N/A | N/A | 1.00 (ref) | **<0.001** |
| Some pumping | N/A |  | N/A |  | 0.25 (0.15-0.41) |  |

^a^ Sample size for unadjusted model n=352

^b^ Sample size for adjusted models n=311

^c^ Model adjusted for maternal education, parity, single parenting, geographic location, household income, and age

^d^ Hosmer Lemeshow test p=0.478

^e^ Model adjusted for same variables as Model 1 plus prenatal intended mode of breast milk delivery

^f^ Hosmer Lemeshow test p=0.782
